# Cost-effectiveness of cannabinoids for pediatric drug-resistant epilepsy: protocol for a systematic review of economic evaluations

**DOI:** 10.1186/s13643-019-0990-z

**Published:** 2019-03-27

**Authors:** Jesse Elliott, Bláthnaid McCoy, Tammy Clifford, Beth K. Potter, Becky Skidmore, George A. Wells, Doug Coyle

**Affiliations:** 1 School of Epidemiology and Public Health, University of Ottawa, 600 Peter Morand Crescent, Ottawa, K1G 5Z3 Canada; 2 Cardiovascular Research Methods Centre, University of Ottawa Heart Institute, 40 Ruskin Street, Ottawa, K1Y4W7 Canada; 3 Department of Paediatrics, University of Toronto, Division of Neurology, The Hospital for Sick Children Toronto, Toronto, Ontario Canada; 4 Independent Information Specialist, Ottawa, Canada

**Keywords:** Cannabis, Cannabidiol, Cost-effectiveness, Pediatric drug-resistant epilepsy, Resource use, Costs, Benefits, Utilities

## Abstract

**Background:**

Drug-resistant epilepsy negatively impacts the quality of life and is associated with increased morbidity and mortality and high costs to the healthcare system. Cannabis-based treatments may be effective in reducing seizures in this population, but whether they are cost-effective is unclear. In this systematic review, we will search for cost-effectiveness analyses involving the treatment of pediatric drug-resistant epilepsy with cannabis-based products to inform decision-making by public healthcare payers about reimbursement of such products. We will also search for cost-effectiveness analyses of other pharmacologic treatments for pediatric drug-resistant epilepsy, as well as estimates of healthcare resource use, costs, and utilities, for use in a subsequent cost-utility analysis to address this decision problem.

**Methods:**

We will search the published and gray literature for economic evaluations of cannabis-based products and other pharmacologic treatments for pediatric drug-resistant epilepsy, as well as resource utilization and utility studies. Two independent reviewers will screen the title and abstract of each identified record and the full-text version of any study deemed potentially relevant. Study and population characteristics, the incremental cost-effectiveness ratio (ICER), as well as total costs and benefits, will be extracted, and quality will be assessed by use of the Drummond and CHEERS checklists; context-specific issues will also be considered. From model-based cost-utility and cost-effectiveness analyses, we will extract and summarize the model structure, including health states, time horizon, and cycle length. From resource utilization studies, we will extract data about the frequency of resource use (e.g., neurology visits, emergency department visits, admissions to hospital). From utility studies, we will extract the utility for each health state, the source of the preferences (e.g., child, parent, patient, general public), and the method of elicitation.

**Discussion:**

Drug-resistant epilepsy in children is associated with important costs to the healthcare system, and decision-makers require high-quality evidence on which to base reimbursement decisions. The results of this review will be useful to both decision-makers considering the decision problem of whether to reimburse cannabis-based products through public formularies and to analysts conducting studies in this area.

**Systematic review registration:**

PROSPERO no.: CRD42018099591.

**Electronic supplementary material:**

The online version of this article (10.1186/s13643-019-0990-z) contains supplementary material, which is available to authorized users.

## Background

Drug-resistant epilepsy (inadequate response to two or more trials of appropriate antiepileptic drugs [1]) affects between 28% and 37% of people with epilepsy [2] and can have profound consequences for affected children and their families, with affected children at increased risk of severe morbidity (e.g., cognitive delay, behavioral problems, autism) and mortality [3]. Pediatric epilepsy is associated with increased contacts with the healthcare system, including neurologist visits, visits to the emergency department, and admissions to hospitals [4, 5], and children with uncontrolled seizures use more health resources than children with controlled seizures, with costs correlated with epilepsy severity [4, 6]. Taken together, resource use by children with epilepsy contributes major costs to the healthcare system [6, 7].

Drug-resistant epilepsy comprises multiple types of pediatric epilepsies, frequently with underlying genetic causes (e.g., Dravet syndrome, Lennox-Gastaut syndrome, West syndrome) [3]. Pediatric epilepsy may also be caused by structural or metabolic (e.g., structural abnormality or chronic metabolic condition) or unknown causes [3]. Because of this variation, and variation in seizure types, there is no standard of care for treatment of pediatric drug-resistant epilepsy, with treatment dependent on the nature of a child’s individual disease, which drugs are available in a given jurisdiction, and local practice [8]. Individual antiepileptic drugs may benefit only small groups of patients because of the rare nature of some epilepsy syndromes, and some antiepileptic drugs have designated orphan drug status (e.g., stiripentol for Dravet syndrome [9]). In addition to pharmacologic treatments (i.e., antiepileptic drugs), other treatment options include non-pharmacologic interventions (e.g., surgery to resect the seizure focus, vagus nerve stimulation, deep brain stimulation therapy, ketogenic diet therapy) [3], with increasing interest in the use of alternative therapies, including cannabinoids [10, 11].

Interest in the use of cannabis for the treatment of pediatric epilepsy has increased over the last decade, with recent clinical studies suggesting that cannabidiol, one of two main cannabinoids in cannabis, reduces seizure burden in some epilepsy syndromes [12, 13]. The first pharmaceutical-grade cannabidiol product intended to treat Dravet syndrome and Lennox-Gastaut syndrome was recently approved by the US Food and Drug Administration [14], and it is expected that public payers will soon be faced with decisions as to whether to include such products on their formularies. Economic evaluations can provide important information to decision-makers as they make such reimbursement and funding decisions [15]. As such, the objective of this systematic review is to provide a comprehensive overview of the cost-effectiveness of cannabis-based treatments for pediatric drug-resistant epilepsy and to search for inputs for a de novo cost-utility analysis to address this decision problem in the Canadian context.

## Research aims

There are three aims of this study:

1. Identify and assess the applicability of existing economic evaluations of cannabis-based products for the treatment of pediatric drug-resistant epilepsy;
2. Identify and assess the suitability of existing models that have been used in the economic evaluation of other pharmacologic treatments in this population;
3. Identify model inputs (e.g., resource use, utilities) from existing economic evaluations of pharmacologic treatments or from stand-alone studies in this population.

The findings of aims 2 and 3 will be used to inform a de novo cost-utility analysis intended to address the decision problem of whether Canadian public healthcare payers should reimburse cannabis-based products for the treatment of drug-resistant epilepsy in children with Dravet syndrome.

## Methods

This systematic review protocol has been registered in PROSPERO (CRD42018099591) and follows the Preferred Reporting Items for Systematic Reviews and Meta-Analyses Protocols (PRISMA-P) statement (Additional file 1) [16]. All screening and data extraction will be performed by the use of standardized and piloted forms in Distiller SR (evidence partners).

### Search strategy

The search strategy was developed in consultation with an experienced medical information specialist and the research team (Additional file 2). The search was peer-reviewed by another senior librarian using the Peer Review of Electronic Search Strategies (PRESS) checklist prior to execution [17]. Databases to be searched include Ovid MEDLINE In-Process and Other Non-Indexed Citations and Ovid MEDLINE and Embase Classic+Embase. The Cochrane Library (HTA database, NHSEED, DSR, DARE, and CENTRAL), Wiley version, will also be searched. The search strategies will be adjusted to the individual database and will include a combination of controlled vocabulary (e.g., Medical Subject Headings) and keywords; no date restrictions will be applied. Gray literature searches will be undertaken using the relevant economic websites and databases listed in Gray Matters [18].

### Eligibility criteria

We will include studies that meet the criteria described below. Conference abstracts and non-English language records will be excluded; however, studies performed in Canada but reported in French will be eligible. Studies that report mixed populations of children and adults will be included if they report data separately for participants aged less than 19 years or if, based on descriptive statistics, we can determine that most patients (i.e., > 80%) are aged less than 19 years.

A) *Research aim 1*

Population: Children (aged less than 19 years) with any form of drug-resistant epilepsy (inadequate response to two or more trials of appropriate antiepileptic drugs [1]).

Intervention: Any type of cannabis or cannabis-based product, cannabinol, cannabidiol (CBD), tetrahydrocannabinol (THC), or combinations of these agents, administered by any route (e.g., oral, inhalation), involving any strain of cannabis and any ratio of THC to CBD.

Comparator: Pharmacologic (i.e., antiepileptic drugs) or non-pharmacologic treatments (i.e., ketogenic diet, epilepsy surgery, vagus nerve stimulation) for pediatric drug-resistant epilepsy.

Study designs: Full economic evaluations (i.e., cost-utility analysis, cost-effectiveness analysis, cost-benefit analysis, cost-minimization analysis) and health technology assessments that include a full economic evaluation. Both trial-based and model-based economic evaluations will be eligible for inclusion.

B) *Research aim 2:*

Population: Children with any form of drug-resistant epilepsy [1].

Interventions and comparators: Pharmacologic treatments for pediatric drug-resistant epilepsy compared with other pharmacologic or non-pharmacologic treatments. Study designs: Full economic evaluations and health technology assessments that involve model-based cost-utility or cost-effectiveness evaluations.

C) *Research aim 3:*

Population: Children with any form of drug-resistant epilepsy (resource utilization, utility, productivity studies) or adult caregivers of children with drug-resistant epilepsy (utility, productivity studies).

Study designs: Resource utilization studies involving at least five children, utility studies, productivity studies; full economic evaluations identified as part of Aim 1 or 2.

### Screening and study selection

Two independent reviewers will screen the title and abstract of each identified record and the full-text version of any study deemed potentially relevant, with disagreements resolved by discussion.

### Outcomes

*Research aim 1*: Total and incremental costs; direct, indirect (i.e., productivity costs), and drug-related costs; total and incremental quality-adjusted life years (QALYs); and incremental cost-effectiveness ratios (ICERs).

*Research aim 2:* Model structure (e.g., description of the included health states, horizon, cycle length), model inputs (e.g., resource use [e.g., number of neurologist visits, admission to hospital, visits to the emergency department] and utility values).

*Research aim 3:* Resource use, costs, and utility values, and by seizure status (e.g., controlled or uncontrolled seizures). From resource utilization studies, the number of resource units used (e.g., number of hospital admissions) will be extracted from all studies; associated costs will be extracted only from studies performed in Canada. Depending on the perspective, costs may include those incurred by the payer (e.g., costs to the healthcare system) or to society (e.g., costs to patients and caregivers, productivity costs). Productivity costs may include lost productivity, lost leisure time, and lost income by caregivers of affected children, as well as productivity losses by children with drug-resistant epilepsy. Utilities may be related to either affected children or their caregivers.

### Data extraction

Data will be extracted by one reviewer, and the completeness and accuracy verified by a second reviewer. We will extract study characteristics (e.g., study design, location, decision problem, perspective [i.e., patient, hospital, healthcare, society], funding source), as well as population characteristics (e.g., epilepsy syndrome, age, comorbidities, setting or context) and details about the interventions and comparators. Details about the model structure, including health states, time horizon, and cycle length, will be extracted from model-based cost-effectiveness and cost-utility analyses, as well as model inputs, including resource use and utilities. From utility studies, we will extract the utility value for each health state, as well as details about the source of the utilities (e.g., population characteristics, setting), method of elicitation (e.g., time trade-off, standard gamble, preference-based multi-attribute classification system), and description of the health state. From resource utilization studies, we will extract details about the source of the resource use (e.g., population characteristics, setting, number of participants), and study design, along with the frequency of use of each type of reported service.

### Quality and risk of bias assessment

We will evaluate the quality of the included economic evaluations by use of the 10-item Drummond checklist [19] and the CHEERS statement [20]. We will also evaluate whether the economic evaluations adequately considered context-specific issues, which may influence the ability of an evaluation to address the decision problem. For example, this may include how the evaluations accounted for changes to treatment regimens or patient weight gain during the analysis horizon. Each study will be appraised independently by two reviewers, with conflicts resolved by discussion.

### Synthesis

The extracted data, including study and patient characteristics and study quality, will be summarized descriptively. Depending on the study type, model characteristics, resource use, costs, QALYs, and cost-effectiveness results (e.g., ICER) will be summarized. To facilitate comparison between studies, all costs will be reported in 2018 Canadian dollars. First, if necessary, costs will be converted to Canadian Dollars using purchasing power parity for the year of each study and then adjusted to 2018 based on the Bank of Canada Inflation calculator. Where possible, data will be reported separately by drug-resistant epilepsy syndrome and by seizure status (e.g., controlled, uncontrolled).

## Discussion

Epilepsy is a major public health concern, affecting about 50 million people worldwide [21]. Because drug-resistant epilepsy is associated with important costs to the healthcare system [6, 7], it is crucial that the cost-effectiveness of new and existing treatments be evaluated using the best available evidence. This review will provide a comprehensive overview of the cost-effectiveness of current pharmacologic treatments, including cannabis-based treatments, for drug-resistant epilepsy in children, and will serve to inform the subsequent development of a de novo cost-effectiveness analyses in this population.

## Additional files

Additional file 1: PRISMA-P checklist. (PDF 136 kb)

Additional file 2: Search strategy. (PDF 129 kb)

## Abbreviations

CBD: Cannabidiol
ICER: Incremental cost-effectiveness ratios
QALY: Quality-adjusted life year
THC: Tetrahydrocannabinol
