# Supplementary material for: Cost-effectiveness of cannabinoids for pediatric drug-resistant epilepsy: protocol for a systematic review of economic evaluations
Source: Syst Rev. 2019 Mar 27;8:75. doi: 10.1186/s13643-019-0990-z (PMC6436234; doi:10.1186/s13643-019-0990-z)
Supplement: Supplementary file 2 — Search strategy. (PDF 129 kb) [file 13643_2019_990_MOESM2_ESM.pdf]

Supplementary File 2:

Elliott et al. Cost-effectiveness of cannabinoids for pediatric drug-resistant epilepsy: protocol for a systematic review of economic evaluations

Ovid Multifile

Database: Embase Classic+Embase <1947 to 2018 June 11>, Ovid MEDLINE(R) ALL <1946 to June 11, 2018>
Search Strategy:

----

1 exp Epilepsy/ (337212)
2 (epileps* or epilept*).tw,kf. (314061)
3 seizure*.tw,kf. (277032)
4 convulsi*.tw,kf. (66672)
5 falling sickness*.tw,kf. (55)
6 comitial disease*.tw,kf. (3)
7 (petit mal or grand mal or absence status).tw,kf. (7552)
8 Landau-Kleffner Syndrome*.tw,kf. (1004)
9 Lennox Gastaut Syndrome*.tw,kf. (3116)
10 Dravet Syndrome*.tw,kf. (1866)
11 West syndrome*.tw,kf. (2669)
12 Doose syndrome*.tw,kf. (157)
13 Ohtahara syndrome*.tw,kf. (459)
14 Sturge-Weber Syndrome/ (3570)
15 ((sturge* or weber) adj2 (disease* or syndrome*)).tw,kf. (7823)
16 (myoclonic encephalopath* or action myoclonus-renal failure syndrome* or atypical inclusion-body disease* or biotin-responsive encephalopath* or haw river syndrome* or may white syndrome* or myoclonus-nephropathy syndrome* or naito oyanagi disease*).tw,kf. (732)
17 SMEI.tw,kf. (419)
18 (MERRF or fukuhara disease* or fukuhara disorder* or myoencephalopathy ragged-red fiber disease* or myoencephalopathy ragged-red fibre disease*).tw,kf. (1072)
19 Lafora.tw,kf. (1308)
20 ((Unverricht adj1 Lundborg) or Baltic Myoclonus or Unverricht disease* or Unverricht syndrome*).tw,kf. (679)
21 ((infantile or nodding) adj2 spasm?).tw,kf. (5772)
22 ((flexor or "in flexion") adj2 spasm?).tw,kf. (313)
23 ((lightning or salaam) adj2 attack?).tw,kf. (15)
24 hypsarrhythmi*.tw,kf. (1936)
25 or/1-24 [EPILEPSY] (560609)
26 Adolescent/ (3369665)
27 exp Child/ (4503995)
28 exp Infant/ (2130677)
29 (boy or boys or girl or girls or infant* or infanc* or baby or babies or child* or toddler* or preschool* or pre-school* or school-age* or adolescen* or teen or teens or teenager* or youth or youths or highschool* or high-school*).tw,kf. (4316665)
30 (newborn* or neonat*).tw,kf. (833360)
31 (pediatric* or paediatric*).tw,kf. (776024)
32 or/26-31 (8172802)
33 25 and 32 [PEDIATRIC EPILEPSY] (203882)
34 exp Technology assessment, biomedical/ (23249)
35 (technology assessment* or HTA or HTAs).tw,kf. (17211)
36 health technology assessment.jw. (3141)
37 or/34-36 [HTAs] (37651)
38 33 and 37 [PAEDIATRIC EPILEPSY - HTAs] (71)
39 exp Epilepsy/ec [Economics] (540)
40 Economics/ (257845)
41 exp "Costs and Cost Analysis"/ (538452)

42 Economics, Nursing/ (38348)
43 Economics, Medical/ (44003)
44 Economics, Pharmaceutical/ (10619)
45 exp Economics, Hospital/ (799622)
46 Economics, Dental/ (37552)
47 exp "Fees and Charges"/ (68883)
48 exp Budgets/ (39309)
49 budget*.ti,ab,kf. (59686)
50 (economic* or cost or costs or costly or costing or price or prices or pricing or pharmacoeconomic* or pharmaco-economic* or expenditure or expenditures or expense or expenses or financial or finance or finances or financed).ti,kf. (419450)
51 (economic* or cost or costs or costly or costing or price or prices or pricing or pharmacoeconomic* or pharmaco-economic* or expenditure or expenditures or expense or expenses or financial or finance or finances or financed).ab. /freq=2 (585534)
52 (cost* adj2 (effective* or utilit* or benefit* or minimi* or analy* or outcome or outcomes)).ab,kf. (322118)
53 (value adj2 (money or monetary)).ti,ab,kf. (4814)
54 exp models, economic/ (14513)
55 economic model*.ab,kf. (6529)
56 markov chains/ (15421)
57 markov.ti,ab,kf. (42108)
58 monte carlo method/ (58618)
59 monte carlo.ti,ab,kf. (83017)
60 exp Decision Theory/ (12808)
61 (decision* adj2 (tree* or analy* or model*)).ti,ab,kf. (45992)
62 exp Delivery of health Care/ec [economics] (49499)
63 ((healthcare or health care or resource?) adj3 (utili#ation? or utilise? or utilize? or utili#ing)).ti,ab,kf. (65949)
64 (resource? adj3 ("use" or used or uses or using)).ti,ab,kf. (57658)
65 (resource? adj3 (unit or units)).ti,ab,kf. (1764)
66 (resource? adj3 (value? or valuation?)).ti,ab,kf. (2929)
67 ((utility or utili#ation) adj (study or studies)).ti,ab,kf. (2946)
68 (ICER or ICERs).ti,ab,kf. (11261)
69 or/39-68 [ECONOMICS, COST-UTILITY] (1992079)
70 33 and 69 [PAEDIATRIC EPILEPSY - ECONOMICS, COST-UTILITY] (3310)
71 "Value of Life"/ (135868)
72 Quality of Life/ (553323)
73 quality of life.ti,kf. (155404)
74 ((instrument or instruments) adj3 quality of life).ab. (7082)
75 Quality-Adjusted Life Years/ (31484)
76 quality adjusted life.ti,ab,kf. (26122)
77 (qaly* or qald* or qale* or qtime* or life year or life years).ti,ab,kf. (43237)
78 disability adjusted life.ti,ab,kf. (5604)
79 (daly or dalys).ti,ab,kf. (5292)
80 (sf36 or sf 36 or short form 36 or shortform 36 or short form36 or shortform36 or sf thirtysix or sfthirtysix or sfthirty six or sf thirty six or shortform thirtysix or shortform thirty six or short form thirtysix or short form thirty six).ti,ab,kf. (58225)
81 (sf6 or sf 6 or short form 6 or shortform 6 or sf six or sfsix or shortform six or short form six or shortform6 or short form6).ti,ab,kf. (3888)
82 (sf8 or sf 8 or sf eight or sfeight or shortform 8 or shortform 8 or shortform8 or short form8 or shortform eight or short form eight).ti,ab,kf. (1041)
83 (sf12 or sf 12 or short form 12 or shortform 12 or short form12 or shortform12 or sf twelve or sftwelve or shortform twelve or short form twelve).ti,ab,kf. (12539)

84 (sf16 or sf 16 or short form 16 or shortform 16 or short form16 or shortform16 or sf sixteen or sfsixteen or shortform sixteen or short form sixteen).ti,ab,kf. (82)
85 (sf20 or sf 20 or short form 20 or shortform 20 or short form20 or shortform20 or sf twenty or sftwenty or shortform twenty or short form twenty).ti,ab,kf. (868)
86 (hql or hqol or h qol or hrqol or hr qol).ti,ab,kf. (36071)
87 (hye or hyes).ti,ab,kf. (178)
88 (health* adj2 year* adj2 equivalent*).ti,ab,kf. (97)
89 (pqol or qls).ti,ab,kf. (911)
90 (quality of wellbeing or quality of well being or index of wellbeing or index of well being or qwb).ti,ab,kf. (1165)
91 nottingham health profile*.ti,ab,kf. (2545)
92 sickness impact profile.ti,ab,kf. (2238)
93 exp health status indicators/ (292343)
94 (health adj3 (utilit* or status)).ti,ab,kf. (140789)
95 (utilit* adj3 (valu* or measur* or health or life or estimat* or elicit* or disease or score* or weight)).ti,ab,kf. (26067)
96 Patient Preference/ (20266)
97 (preference* adj3 (valu* or measur* or health or life or estimat* or elicit* or disease or score* or instrument or instruments)).ti,ab,kf. (20490)
98 disutilit*.ti,ab,kf. (1083)
99 rosser.ti,ab,kf. (199)
100 willingness to pay.ti,ab,kf. (10950)
101 standard gamble*.ti,ab,kf. (1818)
102 (time trade off? or time tradeoff?).ti,ab,kf. (3037)
103 (tto or ttos).ti,ab,kf. (2341)
104 (hui or hui1 or hui2 or hui3).ti,ab,kf. (3154)
105 (eq or euroqol or euro qol or eq5d or eq 5d or euroqual or euro qual).ti,ab,kf. (33522)
106 Absenteeism/ (25724)
107 absentee*.ti,ab,kf. (13411)
108 Presenteeism/ (798)
109 presentee*.ti,ab,kf. (2638)
110 productivit*.ti,ab,kf. (108615)
111 ((work* or employ*) adj5 (absenc* or absent* or presenc* or present*)).ti,ab,kf. (235562)
112 ((work* or employ*) adj5 abilit*).ti,ab,kf. (23476)
113 (time adj1 away).ti,ab,kf. (1386)
114 Sick Leave/ (9808)
115 ((sick or medical) adj leave).ti,ab,kf. (10506)
116 or/71-115 [QoL/DISEASE BURDEN] (1571112)
117 33 and 116 [PAEDIATRIC EPILEPSY - QoL/DISEASE BURDEN] (6990)
118 38 or 70 or 117 [HTAs/ECONOMICS/QoL/DISEASE BURDEN] (9727)
119 exp Animals/ not (exp Animals/ and Humans/) (16386162)
120 118 not 119 [ANIMAL-ONLY REMOVED] (6980)
121 120 use medall [MEDLINE RECORDS] (4123)
122 exp epilepsy/ (337212)
123 (epileps* or epilept*).tw,kw. (319442)
124 seizure*.tw,kw. (279367)
125 convulsi*.tw,kw. (67865)
126 falling sickness*.tw,kw. (58)
127 comitial disease*.tw,kw. (3)
128 (petit mal or grand mal or absence status).tw,kw. (7453)
129 Landau-Kleffner Syndrome*.tw,kw. (1041)

130 Lennox Gastaut Syndrome*.tw,kw. (3173)
131 Dravet Syndrome*.tw,kw. (1914)
132 West syndrome*.tw,kw. (2795)
133 Doose syndrome*.tw,kw. (169)
134 Ohtahara syndrome*.tw,kw. (473)
135 sturge-weber syndrome/ (3570)
136 ((sturge* or weber) adj2 (disease* or syndrome*)).tw,kw. (7950)
137 (myoclonic encephalopath* or action myoclonus-renal failure syndrome* or atypical inclusion-body disease* or biotin-responsive encephalopath* or haw river syndrome* or may white syndrome* or myoclonus-nephropathy syndrome* or naito oyanagi disease*).tw,kw. (742)
138 SMEI.tw,kw. (443)
139 (MERRF or fukuhara disease* or fukuhara disorder* or myoencephalopathy ragged-red fiber disease* or myoencephalopathy ragged-red fibre disease*).tw,kw. (1101)
140 Lafora.tw,kw. (1320)
141 ((Unverricht adj1 Lundborg) or Baltic Myoclonus or Unverricht disease* or Unverricht syndrome*).tw,kw. (685)
142 ((infantile or nodding) adj2 spasm?).tw,kw. (5916)
143 ((flexor or "in flexion") adj2 spasm?).tw,kw. (312)
144 ((lightning or salaam) adj2 attack?).tw,kw. (15)
145 hypsarrhythmi*.tw,kw. (1968)
146 or/122-145 [EPILEPSY] (562925)
147 juvenile/ (40218)
148 exp adolescent/ (3369840)
149 exp child/ (4503995)
150 (boy or boys or girl or girls or infant* or infanc* or baby or babies or child* or toddler* or preschool* or pre-school* or school-age* or adolescen* or teen or teens or teenager* or youth or youths or highschool* or high-school*).tw,kw. (4326831)
151 (newborn* or neonat*).tw,kw. (829311)
152 (pediatric* or paediatric*).tw,kw. (796794)
153 or/147-152 (8032752)
154 146 and 153 [PEDIATRIC EPILEPSY] (203319)
155 biomedical technology assessment/ (22142)
156 (technology assessment* or HTA or HTAs).tw,kw. (17805)
157 health technology assessment.jw. (3141)
158 or/155-157 [HTAs] (37034)
159 154 and 158 [PAEDIATRIC EPILEPSY - HTAs] (70)
160 exp epilepsy/ec [Economics] (540)
161 economics/ (257845)
162 cost/ (104904)
163 exp health economics/ (776721)
164 budget/ (36915)
165 budget*.ti,ab,kw. (60026)
166 (economic* or cost or costs or costly or costing or price or prices or pricing or pharmacoeconomic* or pharmaco-economic* or expenditure or expenditures or expense or expenses or financial or finance or finances or financed).ti,kw. (440284)
167 (economic* or cost or costs or costly or costing or price or prices or pricing or pharmacoeconomic* or pharmaco-economic* or expenditure or expenditures or expense or expenses or financial or finance or finances or financed).ab. /freq=2 (585534)
168 (cost* adj2 (effective* or utilit* or benefit* or minimi* or analy* or outcome or outcomes)).ab,kw. (326140)
169 (value adj2 (money or monetary)).ti,ab,kw. (4826)
170 statistical model/ (233242)

171 economic model*.ab,kw. (6749)
172 probability/ (137107)
173 markov.ti,ab,kw. (42814)
174 monte carlo method/ (58618)
175 monte carlo.ti,ab,kw. (84136)
176 decision theory/ (2611)
177 (decision* adj2 (tree* or analy* or model*)).ti,ab,kw. (46752)
178 ((healthcare or health care or resource?) adj3 (utili#ation? or utilise? or utilize? or utili#ing)).ti,ab,kw. (66568)
179 (resource? adj3 ("use" or used or uses or using)).ti,ab,kw. (57797)
180 (resource? adj3 (unit or units)).ti,ab,kw. (1771)
181 (resource? adj3 (value? or valuation?)).ti,ab,kw. (2947)
182 ((utility or utili#ation) adj (study or studies)).ti,ab,kw. (3047)
183 (ICER or ICERs).ti,ab,kw. (11300)
184 or/160-183 [ECONOMICS, COST-UTILITY] (2176315)
185 154 and 184 [PAEDIATRIC EPILEPSY - ECONOMICS, COST-UTILITY] (3834)
186 socioeconomics/ (134357)
187 exp quality of life/ (585448)
188 quality of life.ti,kw. (181629)
189 ((instrument or instruments) adj3 quality of life).ab. (7082)
190 quality-adjusted life year/ (31484)
191 quality adjusted life.ti,ab,kw. (26277)
192 (qaly* or qald* or qale* or qtime* or life year or life years).ti,ab,kw. (43556)
193 disability adjusted life.ti,ab,kw. (5618)
194 (daly or dalys).ti,ab,kw. (5370)
195 (sf36 or sf 36 or short form 36 or shortform 36 or short form36 or shortform36 or sf thirtysix or sfthirtysix or sfthirty six or sf thirty six or shortform thirtysix or shortform thirty six or short form thirtysix or short form thirty six).ti,ab,kw. (58463)
196 (sf6 or sf 6 or short form 6 or shortform 6 or sf six or sfsix or shortform six or short form six or shortform6 or short form6).ti,ab,kw. (3900)
197 (sf8 or sf 8 or sf eight or sfeight or shortform 8 or shortform 8 or shortform8 or short form8 or shortform eight or short form eight).ti,ab,kw. (1043)
198 (sf12 or sf 12 or short form 12 or shortform 12 or short form12 or shortform12 or sf twelve or sftwelve or shortform twelve or short form twelve).ti,ab,kw. (12582)
199 (sf16 or sf 16 or short form 16 or shortform 16 or short form16 or shortform16 or sf sixteen or sfsixteen or shortform sixteen or short form sixteen).ti,ab,kw. (82)
200 (sf20 or sf 20 or short form 20 or shortform 20 or short form20 or shortform20 or sf twenty or sftwenty or shortform twenty or short form twenty).ti,ab,kw. (867)
201 (hql or hqol or h qol or hrqol or hr qol).ti,ab,kw. (36232)
202 (hye or hyes).ti,ab,kw. (182)
203 (health* adj2 year* adj2 equivalent*).ti,ab,kw. (100)
204 (pqol or qls).ti,ab,kw. (913)
205 (quality of wellbeing or quality of well being or index of wellbeing or index of well being or qwb).ti,ab,kw. (1173)
206 nottingham health profile/ (394)
207 nottingham health profile*.ti,ab,kw. (2557)
208 sickness impact profile/ (9156)
209 sickness impact profile.ti,ab,kw. (2275)
210 health status indicator/ (24834)
211 (health adj3 (utilit* or status)).ti,ab,kw. (142207)

212 (utilit* adj3 (valu* or measur* or health or life or estimat* or elicit* or disease or score* or weight)).ti,ab,kw. (26188)
213 Patient Preference/ (20266)
214 (preference* adj3 (valu* or measur* or health or life or estimat* or elicit* or disease or score* or instrument or instruments)).ti,ab,kw. (20560)
215 disutilit*.ti,ab,kw. (1086)
216 rosser.ti,ab,kw. (200)
217 willingness to pay.ti,ab,kw. (11074)
218 standard gamble*.ti,ab,kw. (1838)
219 (time trade off? or time tradeoff?).ti,ab,kw. (3066)
220 (tto or ttos).ti,ab,kw. (2352)
221 (hui or hui1 or hui2 or hui3).ti,ab,kw. (3168)
222 (eq or euroqol or euro qol or eq5d or eq 5d or euroqual or euro qual).ti,ab,kw. (33591)
223 absenteeism/ (25724)
224 absentee*.ti,ab,kw. (13617)
225 presenteeism/ (798)
226 presentee*.ti,ab,kw. (2699)
227 productivit*.ti,ab,kw. (109229)
228 ((work* or employ*) adj5 (absenc* or absent* or presenc* or present*)).ti,ab,kw. (235647)
229 ((work* or employ*) adj5 abilit*).ti,ab,kw. (23541)
230 (time adj1 away).ti,ab,kw. (1389)
231 medical leave/ (5661)
232 ((sick or medical) adj leave).ti,ab,kw. (10652)
233 or/186-232 [QoL/DISEASE BURDEN] (1345906)
234 154 and 233 [PAEDIATRIC EPILEPSY - QoL/DISEASE BURDEN] (5407)
235 159 or 185 or 234 [HTAs/ECONOMICS/QoL/DISEASE BURDEN] (8755)
236 animal/ or exp animal experimentation/ or exp animal model/ or exp animal experiment/ or nonhuman/ or exp vertebrate/ (48151623)
237 exp human/ or exp human experimentation/ or exp human experiment/ (37022786)
238 236 not 237 (11130520)
239 235 not 238 [ANIMAL-ONLY REMOVED] (8679)
240 conference abstract.pt. (3034537)
241 239 not 240 [CONFERENCE ABSTRACTS REMOVED] (7205)
242 241 use emczd [EMBASE RECORDS] (4490)
243 121 or 242 [BOTH DATABASES] (8613)
244 limit 243 to yr="2010-current" (4067)
245 remove duplicates from 244 (3064)
246 243 not 244 (4546)
247 remove duplicates from 246 (3682)
248 245 or 247 [TOTAL UNIQUE RECORDS] (6746)
249 248 use medall [MEDLINE UNIQUE RECORDS] (4087)
250 248 use emczd [EMBASE UNIQUE RECORDS] (2659)
